# Supplementary material for: Structure and nature of ice XIX
Source: Nat Commun. 2021 May 26;12:3162. doi: 10.1038/s41467-021-23399-z (PMC8155070; doi:10.1038/s41467-021-23399-z)
Supplement: Supplementary file 1 — Supplementary Information [file 41467_2021_23399_MOESM1_ESM.pdf]

Supplementary Information

Structure and nature of ice XIX

Christoph G. Salzmann,*[1] John S. Loveday,[2] Alexander Rosu-Finsen,[1] Craig L. Bull[3]

[1] *Department of Chemistry, University College London, 20 Gordon Street, London WC1H 0AJ, U.K.*

[2] *School of Physics and Astronomy and Centre for Science at Extreme Conditions, University of Edinburgh, Edinburgh EH9 3JZ, U.K.*

[3] *ISIS Neutron and Muon Facility, Rutherford Appleton Laboratory, Didcot, OX11 0QX, U.K.*

email: c.salzmann@ucl.ac.uk

Supplementary note 1

<u>Fitting the ice XIX diffraction data with the *P*4$_2$/*nmc* ice VI crystallographic model:</u> Supplementary Figure 1(left) shows the Rietveld fit of the low-temperature ice XIX diffraction data using the ice VI crystallographic model. Most of the Bragg peaks are fitted quite well. However, the ice VI model does not allow the two additional Bragg peaks at ~2.14 and ~2.21 Å, and the intensity of the Bragg peak at ~1.77 Å is too low. Due to a combination of the symmetry of *P*4$_2$/*nmc* and the constraints imposed by the ice rules, the ice VI model does not allow any deviation of the fractional occupancies of the hydrogen sites from ½ which can therefore not be refined. The structure of ice VI obtained from the Rietveld fit displays realistic bond distances and angles as shown in Supplementary Figure 1(right).

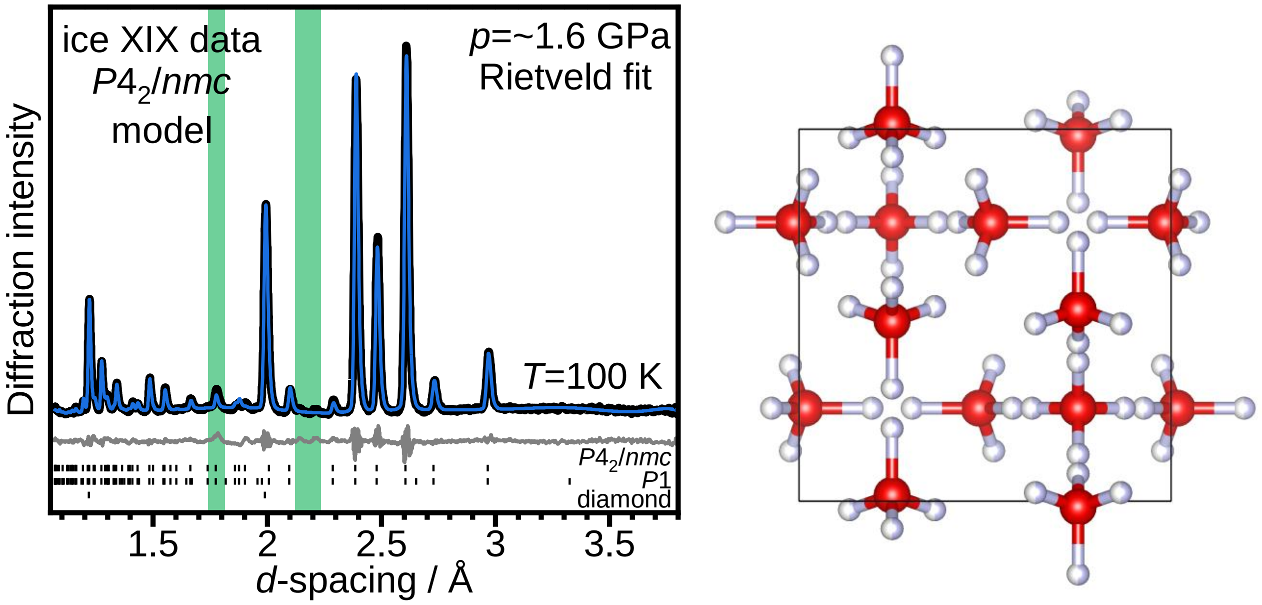

Supplementary Figure 1. *(left) Fitting the low-temperature diffraction data with the P4$_2$/nmc ice VI model. In addition to the P4$_2$/nmc tickmarks, the P1 tickmarks for the same-size unit cell are included. The regions with diffraction features characteristic for ice XIX are highlighted in light green. (right) The ice VI crystal structure obtained from the Rietveld fit.*

These results indicate that the crystal structures of ices VI and XIX are not fundamentally different. However, the inability of the ice VI model to reproduce the additional Bragg peaks at ~2.14 and ~2.21 Å shows that a new crystallographic model is needed for ice XIX.

Supplementary Figure 1(left) also shows the expected peak positions for a corresponding *P*1 unit cell with the same lattice constants as ice VI which also does not permit the additional Bragg peaks. This therefore indicates that the size of the unit cell needs to be increased. Indexing the Bragg peaks with the *dicvol06* software[1] suggests a √2×√2×1 supercell of the ice VI unit cell. According to this, the new peaks are indexed as 231 and 321 for the ~2.14 Å feature and 212 for the ~2.21 Å peak. As shown in the next section, this specific increase in the size of the unit cell arises frequently as the symmetry of the *P*4$_2$/*nmc* space group is reduced to its various crystallographic subgroups.

Supplementary note 2

Search for relevant crystallographic subgroups of *P*4$_2$/*nmc*: The crystallographic subgroups the *P*4$_2$/*nmc* space group were investigated systematically. Our approach was to investigate the first two levels of subgroups which leads to either 3 or 4 distinct oxygen sites through Wyckoff splitting. Ice VI contains two distinct oxygen positions, which are labelled as 'waist' and 'apex' in Figure 1c. Based on the appearance of the additional Bragg peaks, only subgroup pathways leading to √2×√2×1 unit cells were considered. The resulting structures were then analysed with respect to two criteria: (1) If they allow the new Bragg peaks, which is not the case for all √2×√2×1 unit cells, and (2) if the two hydrogen-bonded networks contain the same number of atom sites. As shown in Table S1, this analysis leads to six possible candidate space groups (*P4$_2$nm, P4$_2$cm, Pcnb, Pcca, Pnna* and *Pncb*). Please note that *Pcnb* found here and the *Pbcn* setting in the main article are only different settings of space group 60 and hence describe the same structure.

Supplementary Table 1*. First two levels of crystallographic subgroups of P4$_2$/nmc that lead to √2×√2×1 supercells (highlighted in bold). For the 2nd level subgroups, it is indicated if the new Bragg peaks are permitted and if the structures contain the same number of atom sites for each of the two networks. The structures shown in Figure 3 in the main article are highlighted with asterisks.*

1st level subgroup	2nd level subgroup	new Bragg peaks?	same number of sites per network?
P-4*m*2 (#115)	***C*222 (#21)**	NO	YES
P-4*m*2 (#115)	***P*-42$_1$*m* (#113)**	YES	NO
P-4*m*2 (#115)	***P*-42*m* (#111)**	YES	NO
P-42$_1$*c* (#114)	***C*222 (#21)**	NO	YES
*P*4$_2$*mc* (#105)	***C*222 (#21)**	NO	YES
*P*4$_2$*mc* (#105)	***P*4$_2$*nm* (#102)**	YES	YES
*P*4$_2$*mc* (#105)	***P*4$_2$*cm* (#101)**	YES	YES
*P*4$_2$2$_1$2 (#94)	***C*222 (#21)**	NO	YES
*P*4$_2$/*n* (#86)	none		
Pmmn (#59)	none		
***Ccca* (#68)**	***C2cb (#41)***	NO	YES
***Ccca* (#68)**	***Cc2a (#41)***	NO	YES
***Ccca* (#68)**	***Ccc2 (#37)****	NO	YES
***Ccca* (#68)**	***C222 (#21)***	NO	YES
***Ccca* (#68)**	***C2/c (#15)***	NO	YES
***Ccca* (#68)**	***P2/a (#13)****	NO	YES
***Ccca* (#68)**	***Pcnb* (#60)***	YES	YES
***Ccca* (#68)**	***Pcca* (#54)**	YES	YES
***Ccca* (#68)**	***Pnna* (#52)***	YES	YES
***Ccca* (#68)**	***Pncb* (#50)**	YES	YES

The only immediate subgroup of *P*4$_2$/*nmc* that yields a √2×√2×1 supercell is *Ccca*. However, this space group does not permit the additional Bragg peaks. The other subgroups of *P*4$_2$/*nmc* retain the same size unit cell as ice VI and therefore also do not permit the additional Bragg peaks. This means that the symmetry needs to be reduced further to the subgroups of the subgroups of *P*4$_2$/*nmc*. It is worth stressing that all candidate space groups shown in Figure 3, which were derived based on crystal-chemical reasoning in the main article, appear in our systematic subgroup analysis here and are highlighted by asterisks in Table S1. The *P*2/*a* structure identified here is a different setting describing the same structure as the structure with the

conventional *P*2/*c* setting used in the main article. As mentioned earlier, the same applies to the *Pcnb* setting derived here and *Pbcn* in the main article. The two structures differ only in the definitions of the crystallographic axes. The six possible candidate structures resulting from this systematic subgroup analysis are shown in Supplementary Figure 2.

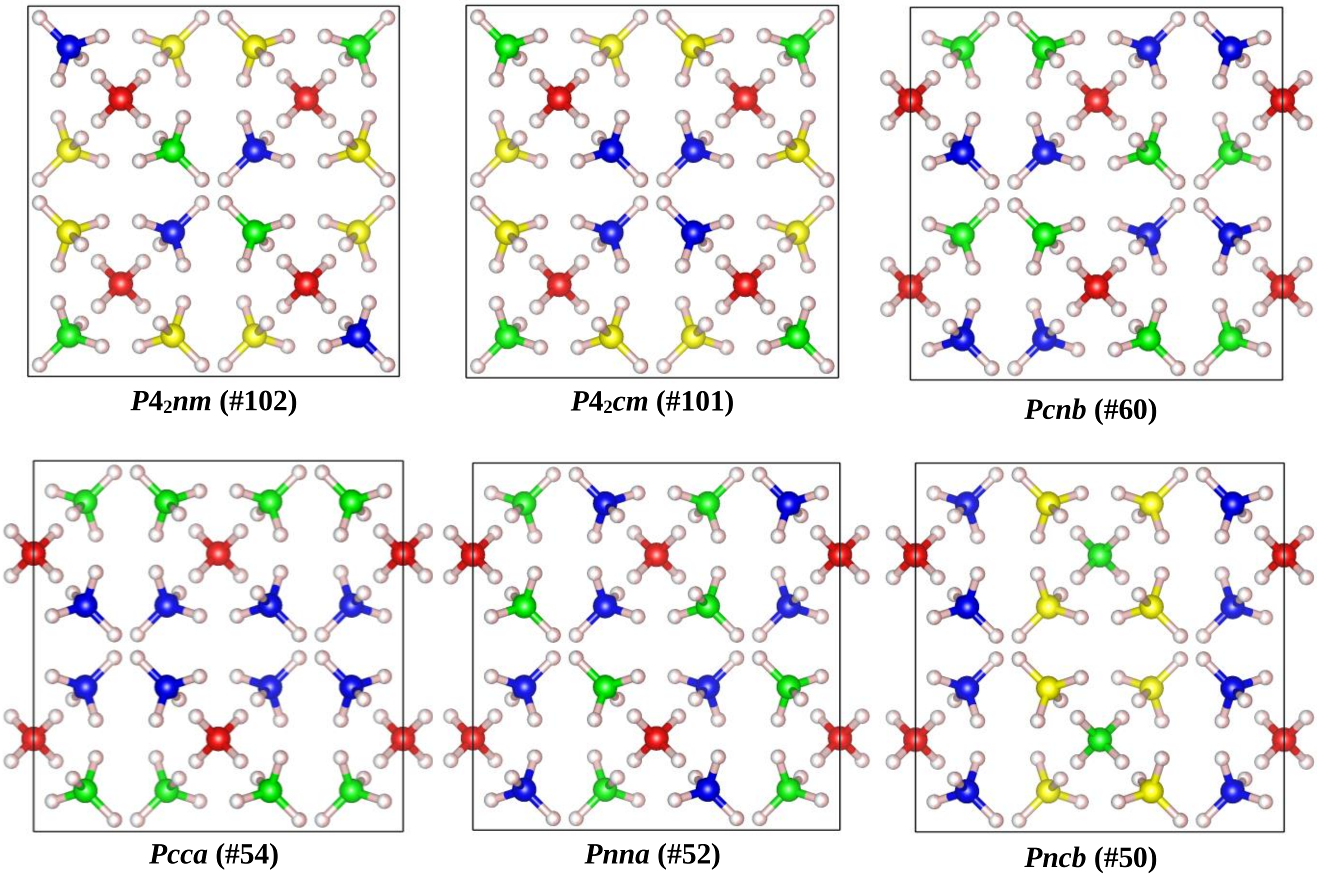

Supplementary Figure 2. *Six candidate structures with space group symmetries derived from the group – subgroup analysis. Larger spheres represent oxygen and smaller spheres hydrogen atoms. The different oxygen sites are indicated by different colours.*

In the $P4_2nm$ and $P4_2cm$ structures, two of the waist oxygen positions remain of the same type whereas the other two become distinct positions. Intuitively speaking, this seems somewhat unusual. Furthermore, these two structures are polar with 4*mm* point group symmetry.

The four possible subgroup structures of *Ccca* are all non-polar. In the *Pncb* structure, two neighbouring hexameric units of the same network become entirely symmetry independent from one another. Since the neighbouring hexameric units have identical structures in ice VI, it seems likely that they will retain at least some symmetry relationship between them in ice XIX which is in contraction with the *Pncb* model. Despite the symmetry concerns, all six candidate structures were fitted against the experimental diffraction data in a next step together with the *Ccca* structure which seems to be an important first-level subgroup of $P4_2/nmc$.

Supplementary note 3

Tests of the subgroup candidate structures: Supplementary Figure 3 shows the χ^2 values, which reflect the goodness of fit, obtained upon fitting the ice VI $P4_2/nmc$ crystallographic model (see chapter 1), the first-level subgroup *Ccca* structure and the six second-level subgroup structures to the experimental ice XIX diffraction data. All structures were fully hydrogen disordered which is strictly required from a combination of symmetry and the ice rules for $P4_2/nmc$ and *Ccca*. Despite being unable to reproduce the additional Bragg peaks (see Table S1), the *Ccca model* could already fit the intensity of the Bragg peak at ~1.77 Å well which led to an overall better fit compared to the $P4_2/nmc$ model. The *Pcnb* model (which is **equivalent** to the *Pbcn* model discussed in the main article) gave the best fit to the data out of all the candidate structures. This means that both the crystal-chemical considerations presented in the main article as well as the systematic group-subgroup investigations carried out here point towards this crystallographic model. Despite the symmetry concerns raised earlier, the second best fit is provided by the $P4_2$nm model.

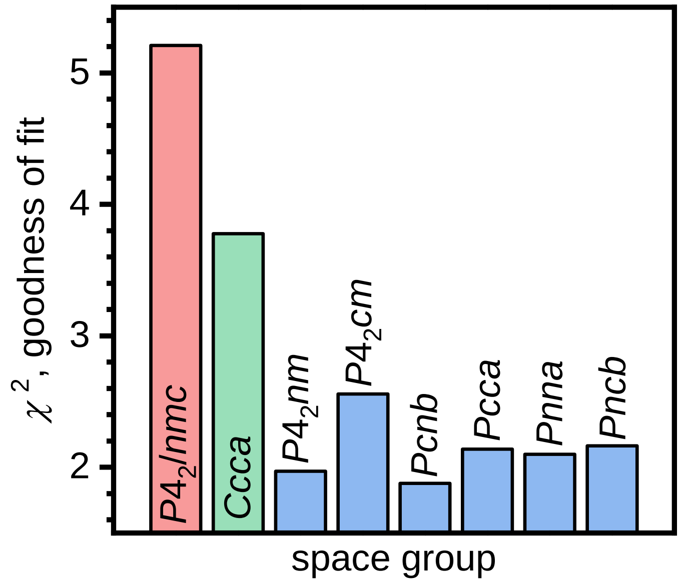

Supplementary Figure 3. *Goodness-of-fit values, χ^2, obtained upon fitting the $P4_2/nmc$ ice VI crystallographic model, the first subgroup level Ccca model and the six second-level subgroup structures to the experimental ice XIX diffraction data. All structures were fully hydrogen disordered which is strictly required from combinations of the symmetry and the ice rules for the $P4_2/nmc$ and Ccca structures.*

Supplementary note 4

Symmetry analysis of the RMCProfile supercell structures: In addition to the 'top-down' subgroup approach for searching for the space group symmetry of ice XIX, we also performed a 'bottom-up' strategy by searching for symmetry in the large supercells resulting from the RMCProfile[2] analyses of both the ice VI and the ice XIX data. The supercells were 'projected' into the √2×√2×1 cell and the average positions of the various atoms were calculated. Due to the quite diffuse hydrogen positions in both structures, this analysis focussed on the oxygen positions. The average positions were analysed for symmetry by using the

FINDSYM software.[3] Within FINDSYM, it is possible to specify a 'search distance' which defines the tolerance with respect to the atomic positions when searching for symmetry. For search distances of 0 Å, the resulting space group was *P*1 for both structures. This is not surprising since a structure reconstruction with RMCProfile cannot be expected to precisely deliver the high symmetry structure. Upon increasing the search distance, the space group *Pm* was found first for ice VI and then the expected $P4_2/nmc$ space group above search distances of 0.09 Å as shown in Supplementary Figure 4. The symmetry analysis of the RMCProfile model of ice XIX revealed a variety of space groups upon increasing the search distance. Above 0.36 Å, *Pbcn* was identified consistent with the earlier analyses. Remarkably, the sequence of space groups seen for smaller search distances corresponds to the sequence of subgroups of *Pbcn* discussed in the main article.

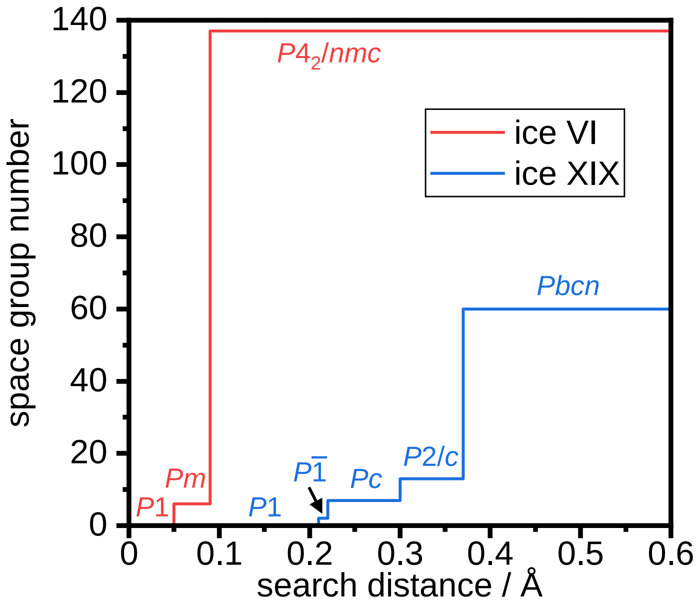

Supplementary Figure 4. *Space group analysis of the RMCProfile supercells using the FINDSYM software.[3] The identified space group numbers are given as a function of the search distance.*

Supplementary note 5

Full refinement of the fractional occupancies using the *Pcnb* / *Pbcn* model: To address the question of hydrogen order present in ice XIX, we performed a full Rietveld refinement of the *Pcnb* model against the ice XIX diffraction data including refinements of the fractional occupancies of the deuterium sites. The *Pcnb* model was derived above using group - subgroup relationships and corresponds to the *Pbcn* model used in the main article. The ice rules were implemented using linear constraints and chemical composition restraints in GSAS. In addition to the fractional occupancies, the refinement included the lattice constants, atomic coordinates, thermal displacement parameters, background function, peak profile parameters and the scale factor. The obtained occupancies are shown in Supplementary Figure 5 for the various hydrogen sites. The deviations from full hydrogen disorder, which corresponds to a fractional occupancy of ½, are overall quite small. The average absolute deviation from ½ is 0.052. In our experience, the margins of error for fractional occupancies given by GSAS as shown in Supplementary Figure 5 are likely to be underestimates. To obtain a more reliable measure for the errors, the *Pcnb* model was refined against the ice VI diffraction data in a

next step. A lower symmetry model can of course always be used to describe higher symmetry (but not the other way round). The resulting occupancies are also shown in Supplementary Figure 5. The average absolute deviation from ½ was found to be 0.032 in this case. Based on this analysis, it can be concluded that the amount of hydrogen order in ice XIX is very small if it is present at all. An upper limit for the average absolute deviation can be estimated as 0.052 – 0.032 = 0.020. Ice XIX is certainly far from being more ordered than ice XV as it has been claimed.[4] For ice XV, the average deviation of the occupancies from ½ was found to be 0.275.[5] It is emphasised that these conclusions were also reached based on spectroscopic data in ref. 6. Weak hydrogen ordering is compatible and even required by our deep-glassy ice scenario.[6,7]

Regarding the question of hydrogen ordering, it is interesting to point out that the *Ccca* subgroup of *P*4$_2$/*nmc* does not allow any hydrogen ordering. This only becomes possible for the *Pbcn* subgroup of *Ccca*. However, *Ccca* already allows tilting distortions of the hexameric units. This suggests that distortions are indeed the origin of the symmetry breaking and hence the ice VI to ice XIX phase transition.

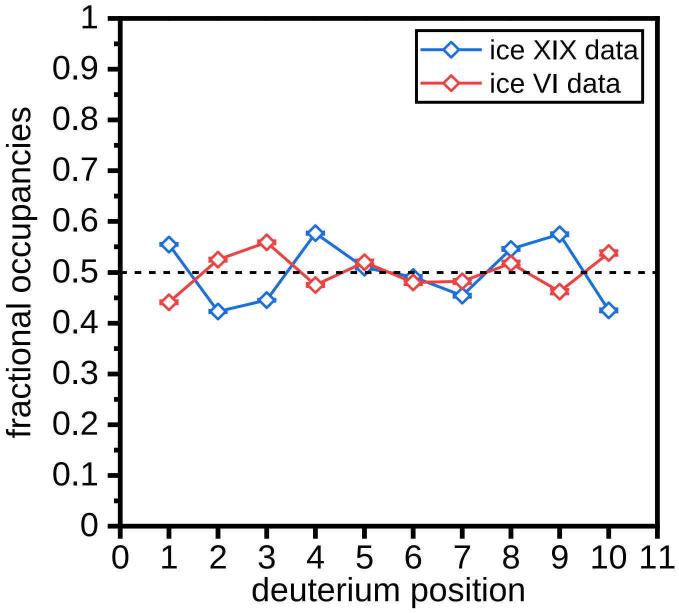

Supplementary Figure 5. *Fractional occupancies of the hydrogen sites obtained using a full Rietveld refinement of the Pcnb model against both the ice XIX and the ice VI diffraction data. The black dashed line indicates full hydrogen disorder.*

Supplementary references

1 Boultif, A. & Louer, D. Program for the Automatic Indexing of Powder Diffraction Patterns by the Successive Dichotomy Method. *J. Appl. Crystal.* **37**, 724-731 (2004).

2 Tucker, M. G., Keen, D. A., Dove, M. T., Goodwin, A. L. & Hui, Q. RMCProfile: reverse Monte Carlo for polycrystalline materials. *J. Phys. Condens. Matter* **19**, 335218 (2007).

3 Stokes, H. T. & Hatch, D. M. Program for Identifying the Space Group Symmetry of a Crystal. *J. Appl. Crystal.* **38**, 237-238 (2005).

4 Gasser, T. M. *et al.* Experiments Indicating a Second Hydrogen Ordered Phase of Ice VI. *Chem. Sci.* **9**, 4224-4234 (2018).

5 Salzmann, C. G. *et al.* Detailed Crystallographic Analysis of the Ice VI to Ice XV Hydrogen Ordering Phase Transition. *J. Chem. Phys.* **145**, 204501 (2016).

6 Rosu-Finsen, A., Amon, A., Armstrong, J., Fernandez-Alonso, F. & Salzmann, C. G. Deep-Glassy Ice VI Revealed with a Combination of Neutron Spectroscopy and Diffraction. *J. Phys. Chem. Lett.* **11**, 1106-1111 (2020).

7 Rosu-Finsen, A. & Salzmann, C. G. Origin of the Low-temperature Endotherm of Acid-doped Ice VI: New Hydrogen-ordered Phase of Ice or Deep Glassy States? *Chem. Sci.*, 515-523 (2019).
